# Rates of Reactions as a Mathematical Consequence of the Permanence of Atoms and the Role of Independent Reactions in the Description of Reaction Kinetics

**DOI:** 10.3389/fchem.2018.00287

**Published:** 2018-07-27

**Authors:** Miloslav Pekař

**Affiliations:** Faculty of Chemistry, Institute of Physical and Applied Chemistry and Materials Research Centre, Brno University of Technology, Brno, Czechia

**Keywords:** kinetics, reaction rate, independent reactions, dimensionality reduction, stoichiometry

## Abstract

Linear algebra treatment of the permanence of atoms (mass conservation) naturally leads to the transformation of formation or destruction rates of components of a reaction mixture into rates of reaction steps, which are sufficient to describe the transformations mathematically. These steps form a scheme of independent reactions that can provide a rational basis for elucidating the reaction mechanism (network) while reducing both the component and parametric dimensionality of the description of kinetics. Several particular reaction examples are used to explain the method and show that rates of additional, dependent reactions cannot be unambiguously related to measured component rates. They also illustrate how the rates of dependent reactions can be correctly expressed in terms of the rates of independent reactions. The method starts only with a knowledge of the components of a reaction mixture. It is argued that the design of consistent reaction networks or mechanisms should take into account not only chemistry but also mathematics.

## Introduction

Chemical kinetics is one of chemistry's disciplines in which mathematics establishes vivid and important applications. This refers not only to the evaluation of kinetic data but also to general theories, both on the molecular (microscopic) and phenomenological (macroscopic) levels. For several decades, mathematicians have known that many empirically established concepts in stoichiometry and mass-action kinetics can be given a formal representation in terms of linear algebra. Surprisingly, this rigorous treatment of physicochemical problems has been largely sidelined in the physicochemical community (in contrast to the area of chemical reaction engineering, where more effort to felicitously apply mathematics in the design of chemical plants can be observed). This is probably due to a lack of practical examples showing the benefits (consequences) of such mathematics for kinetics. To better explain the motivation for this work, we will start with a brief, and only apparently superfluous, discussion of the elementary concepts of chemical kinetics.

The rate of chemical reaction is an established and widely used quantity, though, in fact, it cannot be measured. The rate of chemical reaction is obtained from the amounts (concentrations) of the components of the reaction mixture. These are measured either as a function of time, or, in the stationary state of flow-through systems, for specific values of quantities determining the steady state (e.g., flow rate). Component rates, obtained in this way, are then translated into the language of reaction rates. Also, definitions of the rate of chemical reaction are based on component rates. Let us test these statements by doing a random search of selected textbooks on physical chemistry, chemical kinetics, and reaction engineering.

Silbey et al. (2005) define the rate of a reaction by means of the extent of the reaction, which, in turn, is defined through the change in the amount of any component of the reaction mixture. They state that “the rates of chemical reactions are obtained from measurements of concentration as a function of time.”

A similar definition of the rate of reaction is used by Berry et al. (2000). They stress that the concentrations of the substances taking part in a reaction are the principal variables which affect reaction rates. Further, they state that “the rate of change of a given species is always the sum of the rates of all the processes involving it” and “one must usually know (or guess) what all the significant reactions are, and make some measurements distinguishing them.” They also claim that “the rate at which [B] changes is the algebraic sum of all the rates involving species B” ([B] meaning the concentration of B).

Pilling and Seakins (1996), in fact, do not define the reaction rate. They recall the pioneering study by Wilhelmy on the rate of hydrolysis of sucrose that was expressed as the dependence on the first power of the sucrose concentration. This, and many similar experimental results, are generalized to a rate law in the form of the dependence of the time derivation of the reactant concentration on the product of certain powers of the concentrations of all reactants.

Mortimer and Taylor (2002) claim that “[t]o make progress in understanding the rates of chemical reactions it is necessary to adopt an experimental approach…” They start with a discussion of the rate of change of the concentration of a reactant or product with time and then define the rate of a chemical reaction on this basis—as a concentration time derivative divided by the corresponding stoichiometric coefficient. They also note that this definition assumes constant volume conditions.

Metiu (2006) first indicates that the time derivative of the number of moles would be a natural definition of the reaction rate, but immediately prefers to use the time derivative of the extent of the reaction.

Smith (1981) writes: “The rate of a homogeneous reaction is defined as the change in moles (due to reaction) of a reactant, or product, per unit time per unit volume of the reaction mixture.” From the experimental point of view, this source states that concentration–time profiles are measured and from them rate equations are deduced.

Missen et al. (1999) operate with the extensive rate of reaction with respect to a species A that “is the observed rate of formation of A” (the intensive rate is obtained by referring, for example, to the unit reaction volume or the unit mass of catalyst). For simple (in fact, stoichiometric) reactions, they present the rate of reaction as the component rates divided by the corresponding stoichiometric coefficients. They also note that to measure the reaction rates means to measure the concentrations of the components of the reaction mixture.

Davis and Davis (2003) also define the reaction rate as the time derivative of the extent of the reaction. They stress that this is the definition for a homogeneous, closed system at uniform pressure, temperature, and composition in which a single chemical reaction occurs. They also note that conditions in a chemical reactor are usually quite different from the ideal requirements used in the definition of reaction rates and claim that reaction rates cannot be measured directly in a closed system. In such a system, the composition varies with time and the rate is calculated from these measurements. In flow-through reactors, they consider rate measurements in steady states. In this case, the reaction rate is calculated, in principle, from the fractional conversion, which, in turn, is also determined from the composition, though here it does not change over time.

Thus, the concentrations or concentration changes of individual components, or, at least, of a key component, in a reaction mixture are measured in kinetic experiments. Component rates can be calculated directly from these experiments. A positive component rate means that the respective component is formed during the reaction, whereas a negative component rate indicates that the respective component is consumed. The component rates then serve as a basis for deducing, expressing, and calculating rates of reactions occurring in the reaction mixture. Which reactions occur is usually inferred on the basis of chemical insights, taking into account the structure of detected components (reactants, products, intermediates); here belong also computer modeling studies exploring potential energy surfaces. This part of kinetic research is closely related to the design or discovery of reaction schemes, which can be formulated in the form of a reaction network (a set of suitable, not necessarily elementary, reactions describing chemical transformations in a reaction mixture) or a reaction mechanism (a set of elementary reactions). Once the reactions are designated, their rates can be written in terms of the kinetic mass-action law, i.e., explicitly as a function of the products of rate constants and (the products of) concentrations (raised to powers/partial reaction orders) of species participating in a particular reaction.

It should be realized that component or reaction rates are also mathematical quantities, especially when they are used to calculate or to model, for example, concentration profiles or reactor behavior. They should be consistent with the permanence of atoms or, in other words, with mass conservation. This consistency places some mathematical constraints on the rates considered as mathematical quantities. Using relatively simple methods of linear algebra, Bowen presented a fairly general analysis of such mathematical consequences, starting only with a list of components in a reaction mixture and taking into account also stoichiometric rules (Bowen, 1968). Among others, Bowen showed that reaction rates are mathematical consequences of (the linear algebra of) the permanence of atoms. In other words, the existence of individual chemical reactions and their rates is also a result of the mathematics behind the conservation of atoms (of mass). Consequently, it is not only chemical insights or approaches that can be used to identify reactions occurring in a reaction mixture; mathematics can also give clear guidance in the search for possible reactions. It is the aim of this work to analyze the consequences of linear algebra for the description of reaction kinetics using several explicit examples of chemically reacting mixtures and to make Bowen's fruitful, but still largely unexplored, results more readily available to the chemical community.

## Theoretical methods

For the convenience of readers, the basics of Bowen's analysis (Bowen, 1968; Pekař and Samohýl, 2014) are given in this part. Note that the list of symbols is given at the end.

The conservation of mass in chemical reactions can be written as:

(1) $$\begin{array}{r} \overset{n}{\underset{\alpha =1}{\sum }}r_{w\alpha }=0, \end{array}$$

where *r*_*wα*_ is the mass production rate (per unit volume and unit time) of component α. In fact, mass conservation is due to the conservation of atoms (numbers of each atom) forming the components of the reaction mixture (nuclear reactions are not considered). The conservation can thus be rewritten using the molecular weights *M*_α_ and the molar production rates of components α = 1, 2, …, *n*, *J*^α^, which will henceforth represent the component (formation or consumption) rates:

(2) $$\begin{array}{r} \overset{n}{\underset{\alpha =1}{\sum }}J^{\alpha }M_{\alpha }=0. \end{array}$$

Equation (2) resembles the scalar product of two vectors in the form of their components. Because the product is zero, the two vectors should be perpendicular. These *n*-component vectors are from some *n*-dimensional vector space and can be formally expressed using its basis **e**_α_ and reciprocal basis **e**^α^ as follows.

(3) $$\begin{array}{r} \text{J}=\overset{n}{\underset{\alpha =1}{\sum }}J^{\alpha }{\text{e}}_{\alpha };\;\text{M}=\overset{n}{\underset{\alpha =1}{\sum }}M_{\alpha }{\text{e}}^{\alpha } \end{array}$$

The *n*-dimensional vector space will be called the component space and denoted by U, the vector **J** is called the reaction rate vector, and the vector **M** is called the vector of molar masses. Conservation (2) can also be rewritten using the atomic weights that form the weights of molecules:

(4) $$\begin{array}{r} M_{\alpha }=\overset{z}{\underset{\sigma =1}{\sum }}A^{\sigma }T_{\sigma \alpha }, \end{array}$$

where *A*^σ^ is the weight of (one mole of) atom σ, *T*_σα_is the number of atoms σ in component α, and *z* is the total number of types of atoms. The sum in (2) is

(5) $$\begin{array}{r} \overset{n}{\underset{\alpha =1}{\sum }}J^{\alpha }M_{\alpha }=\overset{z}{\underset{\sigma =1}{\sum }}A^{\sigma }\overset{n}{\underset{\alpha =1}{\sum }}T_{\sigma \alpha }J^{\alpha }. \end{array}$$

Each atom should be conserved; consequently,

(6) $$\begin{array}{r} A^{\sigma }\overset{n}{\underset{\alpha =1}{\sum }}T_{\sigma \alpha }J^{\alpha }=0\Rightarrow \overset{n}{\underset{\alpha =1}{\sum }}T_{\sigma \alpha }J^{\alpha }=0;\;\sigma =1,\;2,\ldots ,z. \end{array}$$

The resulting equality (6) is a homogeneous set of linear algebraic equations for *J*^α^ with matrix ||*T*_σα_||. The component rates are nonzero, i.e., chemical reactions are possible only if the rank (*h*) of this matrix is less than *n*, from which *n*−*h* > 0 follows for a reaction mixture. Generally, only *h* equations are linearly independent; equality (6) can thus be simplified as

(7) $$\begin{array}{r} \overset{n}{\underset{\alpha =1}{\sum }}S_{\sigma \alpha }J^{\alpha }=0;\;\sigma =1,\;2,\ldots ,h, \end{array}$$

where the matrix ||*S*_σα_|| has rank *h* and was created from the matrix *T*_σα_ by the elimination of linearly dependent rows. Molecular weights can then be expressed by:

(8) $$\begin{array}{r} M_{\alpha }=\overset{h}{\underset{\sigma =1}{\sum }}E^{\sigma }S_{\sigma \alpha }, \end{array}$$

where *E*^σ^ is the weight of pseudoatomic element^1^ σ. The vector of molar masses is then:

(9) $$\begin{array}{r} \text{M}=\overset{h}{\underset{\sigma =1}{\sum }}E^{\sigma }\overset{n}{\underset{\alpha =1}{\sum }}S_{\sigma \alpha }{\text{e}}^{\alpha }. \end{array}$$

Because matrix ||*S*_σα_|| has rank *h*, the second sum in (9) represents *h* linearly independent vectors **f**_σ_:

(10) $$\begin{array}{r} {\text{f}}_{\sigma }=\overset{n}{\underset{\alpha =1}{\sum }}S_{\sigma \alpha }{\text{e}}^{\alpha };\;\sigma =1,\;2,\ldots ,h. \end{array}$$

These vectors can thus be considered as the basis of an *h*-dimensional subspace that will be denoted by W. As follows from (9), the vector of molar masses can be expressed in this basis and lies in this subspace:

(11) $$\begin{array}{r} \text{M}=\overset{h}{\underset{\sigma =1}{\sum }}E^{\sigma }{\text{f}}_{\sigma }. \end{array}$$

Subspace W unambiguously determines a complementary orthogonal subspace of dimension *n*−*h* denoted by V. Because **J** .**M** = 0, cf. (2), vector **J** lies in subspace V.

Let us make a preliminary summary of the theoretical background. The vector of molar masses **M** lies in the original component space U but because of the permanence of atoms, i.e., of mass conservation, it is, at the same time, located in its subspace W. Its coordinates in U are the molecular weights, whereas the coordinates in W are the atomic or pseudoatomic weights. The reaction rate vector **J** is also in component space U with coordinates formed by the component rates, cf. (3), and also in subspace V, which is orthogonal to W. The task now is to find the coordinates of **J** in V.

First, the basis of subspace V should be selected. This can be performed as in the case of subspace W, cf. (10), using the base of the original component space:

(12) $$\begin{array}{r} {\text{d}}^{p}=\overset{n}{\underset{\alpha =1}{\sum }}P^{p\alpha }{\text{e}}_{\alpha };p=1,\;2,\ldots ,n-h. \end{array}$$

Here *P*^*pα*^ are elements of a suitable matrix ||*P*^*pα*^|| with rank *n*−*h* and fulfill the orthogonality condition:

(13) $$\begin{array}{r} {\text{f}}_{\sigma }.{\text{d}}^{p}=0\text{ or }||P^{p\alpha }||\cdot {\left||S_{\sigma \alpha }|\right|}^{T}=||0|| \end{array}$$

The coordinates of **J** in V are then generally given by the following relation.

(14) $$\begin{array}{r} \text{J}=\overset{n-h}{\underset{p=1}{\sum }}J_{p}{\text{d}}^{p} \end{array}$$

The meaning of the coordinates *J*_*p*_ follows from the comparison of (14) with (3):

(15) $$\begin{array}{r} \overset{n}{\underset{\alpha =1}{\sum }}J^{\alpha }{\text{e}}_{\alpha }\equiv \overset{n-h}{\underset{p=1}{\sum }}J_{p}{\text{d}}^{p}=\overset{n}{\underset{\alpha =1}{\sum }}\overset{n-h}{\underset{p=1}{\sum }}J_{p}P^{p\alpha }{\text{e}}_{\alpha }. \end{array}$$

From (15) it follows that

(16) $$\begin{array}{r} J^{\alpha }=\overset{n-h}{\underset{p=1}{\sum }}J_{p}P^{p\alpha }. \end{array}$$

Equation (16) shows that the component rates *J*^α^ can be expressed through *J*_*p*_, which, in fact, are the rates of *n*−*h* independent reactions, as indicated by another orthogonality result obtained using (12) and (3):

(17) $$\begin{array}{r} 0={\text{d}}^{p}.\text{M}=\overset{n}{\underset{\alpha =1}{\sum }}P^{p\alpha }M_{\alpha };p=1,\;2,\ldots ,n-h. \end{array}$$

Equations (17) are equations of *n*−*h* chemical reactions if corresponding chemical formulas are introduced instead of *M*_α_; matrix ||*P*^*pα*^|| is then the stoichiometric matrix (the matrix of stoichiometric coefficients) with elements corresponding to the participation of component α in the reaction *p*. Because its rank is *n*−*h*, the reactions determined by (17) are (linearly) independent.

Finally, we can say that the coordinates of the reaction rate vector in subspace V are the rates of independent reactions, *J*_*p*_, which will henceforth be called the reaction rates. Thus, the existence of reaction rates is a consequence of mass conservation based on the permanence of atoms. The reaction rates result from the linear algebraic formulation of the permanence of atoms. If mass conservation is expressed in terms of molecular weights, then the component rates are relevant, cf. (2) and (3). If it is expressed directly in terms of atomic (or pseudoatomic) weights, then the reaction rates follow.

Another consequence of linear algebraic treatment is that only independent reactions are sufficient for describing (mathematically) the transformations (mass or molar amount changes) caused by chemical reactions and measured in kinetic experiments. It should be stressed that the independence of some elements is a mathematical concept and, as such, should be precisely defined. Chemical literature contains descriptions of independent reactions that need not mean the same thing as here. In this work, independence should be understood exactly as expressed by Equation (17) or (16). That is, in the sense of the maximum number of relationships necessary to express the permanence of atoms in terms of molecular weights, none of these relationships can be expressed by a (linear) combination of (some) others; or in the sense of the maximum number of reactions necessary to translate the component rates into the reaction rates in a given reaction mixture, no reaction can be obtained as a (linear) combination of (some) others. The root of this independence lies in the existence of the (*n*−*h*)-dimensional subspace V. Of course, this mathematical analysis does not present an explicit formula for reaction rates (*J*_*p*_). Mass-action expressions, formulated on the basis of experimental experience, are often used in traditional kinetics and can be used also here in (16).

Of the monographs and textbooks referred to above, only that by Missen et al. (1999) provides some basic information on the number of independent chemical reactions (chemical equations in their terminology) and states that “[a] *proper set* of chemical equations for a system is made up of linearly independent equations” and that “stoichiometry tells us the maximum number of independent rate laws that we must obtain experimentally.”

Note that a class of mathematical models of chemical reactions based on atom-free stoichiometry (Érdi and Tóth, 1989) disregards the atomic structure of the reacting chemical compounds. Thus, these models are beyond the scope of the presented methodology. On the other hand, even the real (nonnuclear) reactions in reacting systems described by such models should occur in agreement with the permanence of atoms.

## Results and discussion

It should be stressed that the starting point of the presented analysis is just the composition of a reaction mixture (the determined or supposed reactants, products, and intermediates). Selected examples should be viewed just as examples of this analysis; there is no ambition to discuss or even resolve the potential questions of “true mechanisms” etc.

### Ammonia synthesis

The basic reaction mixture gives simple results. There are three components (N_2_, H_2_, NH_3_; components 1, 2, and 3, respectively) and two atoms (N, H; atoms 1 and 2, respectively) giving the following compositional matrix:

(A1) $$\begin{array}{r} ||T_{\sigma \alpha }||=\left[\begin{array}{ccc} 2 & 0 & 1\\ 0 & 2 & 3 \end{array}\right]. \end{array}$$

Its rank is equal to 2; consequently,||*S*_σα_|| ≡ ||*T*_σα_||. The coordinates of the vector **M** in component space U and in subspace W are, respectively, as follows.

(A2) $$\begin{array}{rcl} \text{M} & = & \left(M_{N_{2}};M_{H_{2}};M_{NH_{3}}\right)\\ \text{M} & = & \left(2A^{N};2A^{H};A^{N}+3A^{H}\right) \end{array}$$

The basis vectors of subspace W are as follows.

(A3) $$\begin{array}{rcl} {\text{f}}_{1} & = & 2{\text{e}}^{1}+{\text{e}}^{3}\\ {\text{f}}_{2} & = & 2{\text{e}}^{2}+3{\text{e}}^{3} \end{array}$$

Here, *n*−*h* = 1and only one independent reaction is possible. Its stochiometric matrix can be selected as follows:

(A4) $$\begin{array}{r} ||P^{p\alpha }||=\left[\begin{array}{ccc} -1 & -3 & 2 \end{array}\right]. \end{array}$$

It is very easy to check that (13)_2_ is fulfilled; the independent reaction is just N_2_+ 3H_2_ = 2NH_3_ and Equation (16) gives:

(A5) $$\begin{array}{r} J^{N_{2}}=-J_{1};\;J^{H_{2}}=-3J_{1};\;J^{NH_{3}}=2J_{1}. \end{array}$$

If additional components of the ammonia synthesis reaction mixture are included, viz. H(ads), N(ads), N_2_(ads), NH(ads), NH_2_(ads), NH_3_(ads) (Pilling and Seakins, 1996), then the compositional matrix ||*T*_σα_|| is of dimension 2 × 9 and its rank is 2. Seven independent reactions are thus possible—this is just the number of reaction steps listed in book by Pilling and Seakins (1996); it is an easy task to check that they are independent reactions.

In the ammonia example, the independent reactions following from the mathematical analysis can be directly identified with the reaction scheme proposed on the chemical basis. Other reaction mixtures need not give such trivial results.

### Atomic chlorine reactions

Pilling and Seakins (1996) present two reactions of chlorine atoms originally generated by chlorofluorocarbons in the stratosphere.

(C1) $$\begin{array}{rcl} \text{Cl}+{\text{O}}_{3} & = & \text{ClO}+{\text{O}}_{2}\\ \text{Cl}+\text{C}{\text{H}}_{4} & = & \text{HCl}+\text{C}{\text{H}}_{3} \end{array}$$

This is a mixture of seven components formed by four atoms. Its composition matrix is of rank 4; thus, three independent reactions are possible. Consequently, the rates of the two reactions (C1) may not be sufficient to describe the kinetics of chemical changes occurring in this mixture. One additional reaction might be added on the condition that (13) is fulfilled. The stoichiometric matrix for such a reaction triple is generally:

(C2) $$\begin{array}{r} \left[\begin{array}{ccccccc} -1 & -1 & 1 & 1 & 0 & 0 & 0\\ -1 & 0 & 0 & 0 & -1 & 1 & 1\\ P^{1} & P^{2} & P^{3} & P^{4} & P^{5} & P^{6} & P^{7} \end{array}\right]; \end{array}$$

(component numbering: 1 = Cl, 2 = O_3_, 3 = ClO, 4 = O_2_, 5 = CH_4_, 6 = HCl, 7 = CH_3_). The orthogonality condition (13) leads to the conclusion that only three of the seven coefficients *P*^*i*^ can be selected independently—e.g., *P*^3^, *P*^4^, *P*^7^in the following example:

(C3) $$\begin{array}{rcl} P^{1} & = & -P^{3}-P^{7};\;P^{2}=\frac{-P^{3}-2P^{4}}{3};\\ P^{5} & = & -P^{7};\;P^{6}=P^{7}. \end{array}$$

Two examples of the mathematically acceptable selection of the third reaction are 2O_3_ = 3O_2_ or 2Cl + O_2_ = 2ClO. Mathematics does not yield a single, unambiguous stoichiometric matrix but only the rules and conditions that any potential stoichiometric matrix should conform to. Chemistry should then be used to select a realistic stoichiometric matrix from the set of those mathematically possible.

### Butene isomerization

Let us further consider a mixture of butene isomers (1,2-; cis-2,3-; trans-2,3-). Here *n* = 3, *z* = 2, and the rank of the matrix:

(B1) $$\begin{array}{r} ||T_{\sigma \alpha }||=\left[\begin{array}{ccc} 4 & 4 & 4\\ 8 & 8 & 8 \end{array}\right] \end{array}$$

is equal to 1, i.e., only two independent reactions exist, whereas three reactions formed by interconversions between each pair of isomers are usually considered. Matrix ||*S*_σα_|| can be selected as [4 4 4], which corresponds to the pseudoatomic element CH_2_ and *E*^1^ = *A*^*C*^ +2*A*^*H*^ (14 g/mol). Another selection could be [1 1 1] with the pseudoatomic element C_4_H_8_. Vector **M** is expressed in the reciprocal basis **e**^α^ as follows (in g/mol)

(B2) $$\begin{array}{r} \text{M}=56{\text{e}}^{1}+56{\text{e}}^{2}+56{\text{e}}^{3} \end{array}$$

and the basis of the 1-dimensional subspace W corresponding to the first selection of the matrix ||*S*_σα_|| is

(B3) $$\begin{array}{r} {\text{f}}_{1}=4{\text{e}}^{1}+4{\text{e}}^{2}+4{\text{e}}^{3}. \end{array}$$

**Figure 1:**
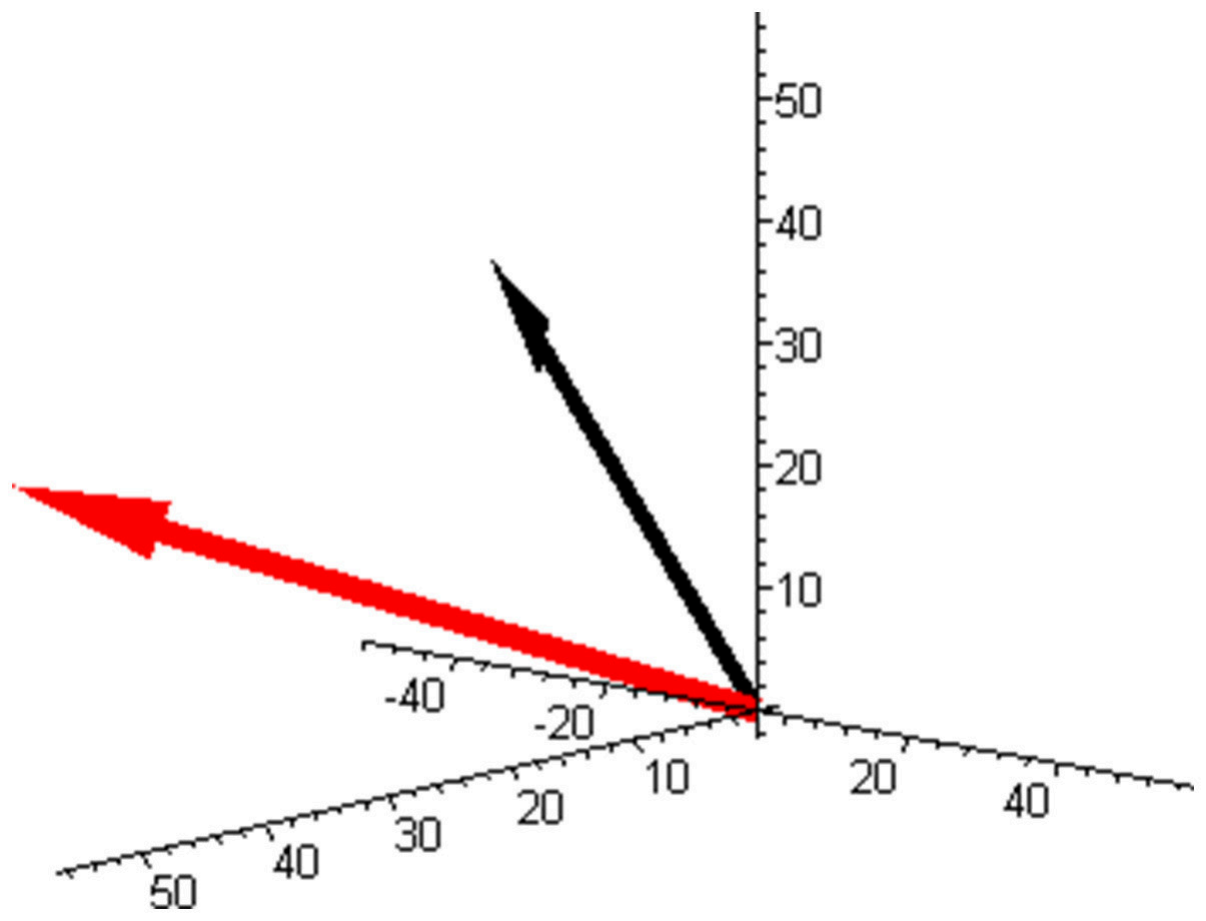
The component space with the molar masses vector (red) and with the example of the reaction rate vector (−50, 30, 20) (black) corresponding to Equation (3) for a mixture of butene isomers (and unit base vectors).

Vector **M** is illustrated in Figure 1 (red arrow). The stoichiometric matrix can be selected as:

(B4) $$\begin{array}{r} ||P^{p\alpha }||=\left[\begin{array}{ccc} -1 & 1 & 0\\ 0 & -1 & 1 \end{array}\right]. \end{array}$$

Component rates are then expressed with the aid of the (independent) reaction rates as follows.

(B5) $$\begin{array}{rcl} J^{1,2} & = & -J_{1}\\ J^{\text{cis}2,3} & = & J_{1}-J_{2}\\ J^{\text{trans}2,3} & = & J_{2} \end{array}$$

An example of the reaction rate vector (3) is also shown in Figure 1 (black arrow).

**Figure 2:**
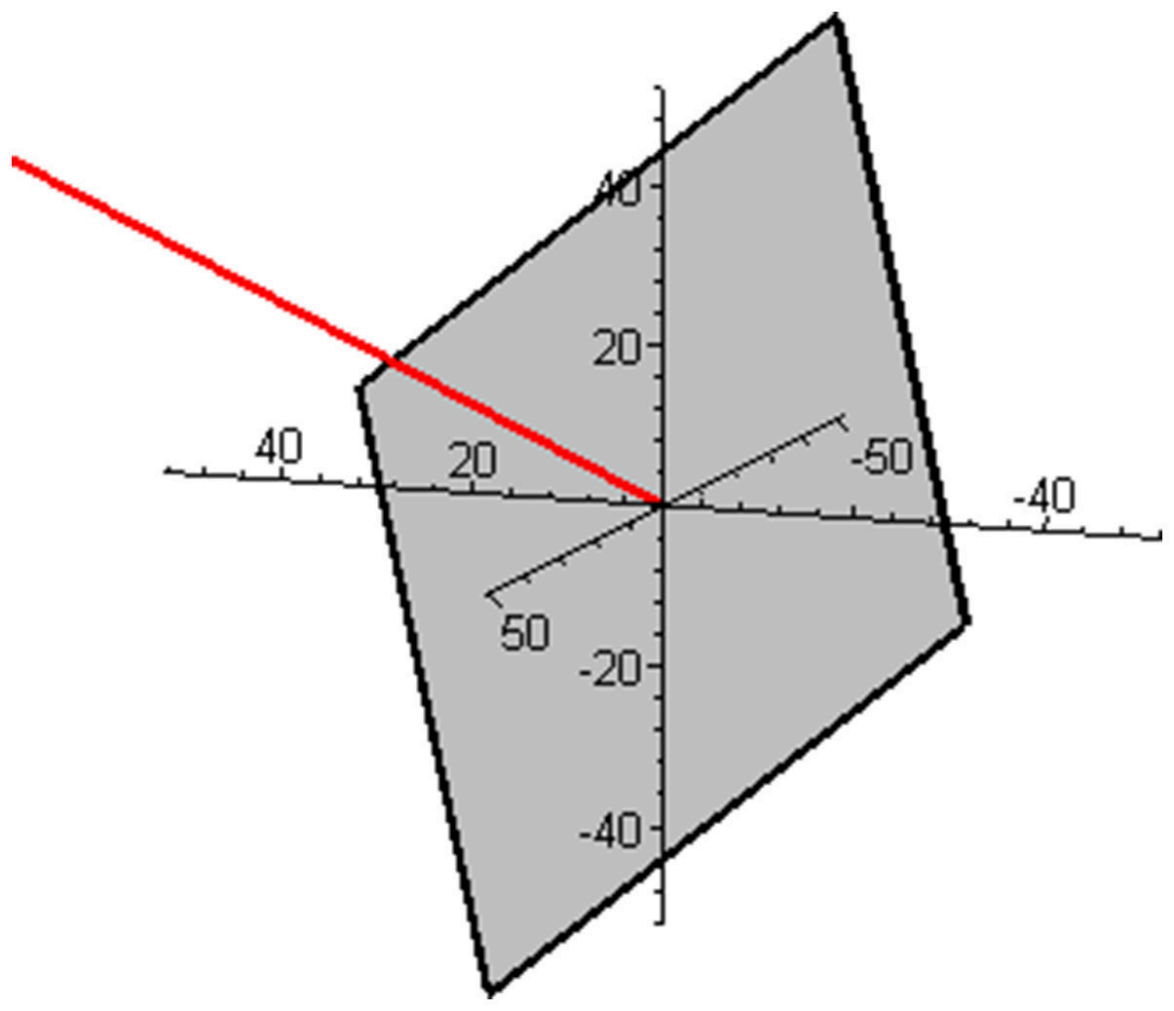
Illustration of the one-dimensional subspace W (red) and the complementary, orthogonal two-dimensional subspace V (gray) in the mixture of butene isomers.

**Figure 3:**
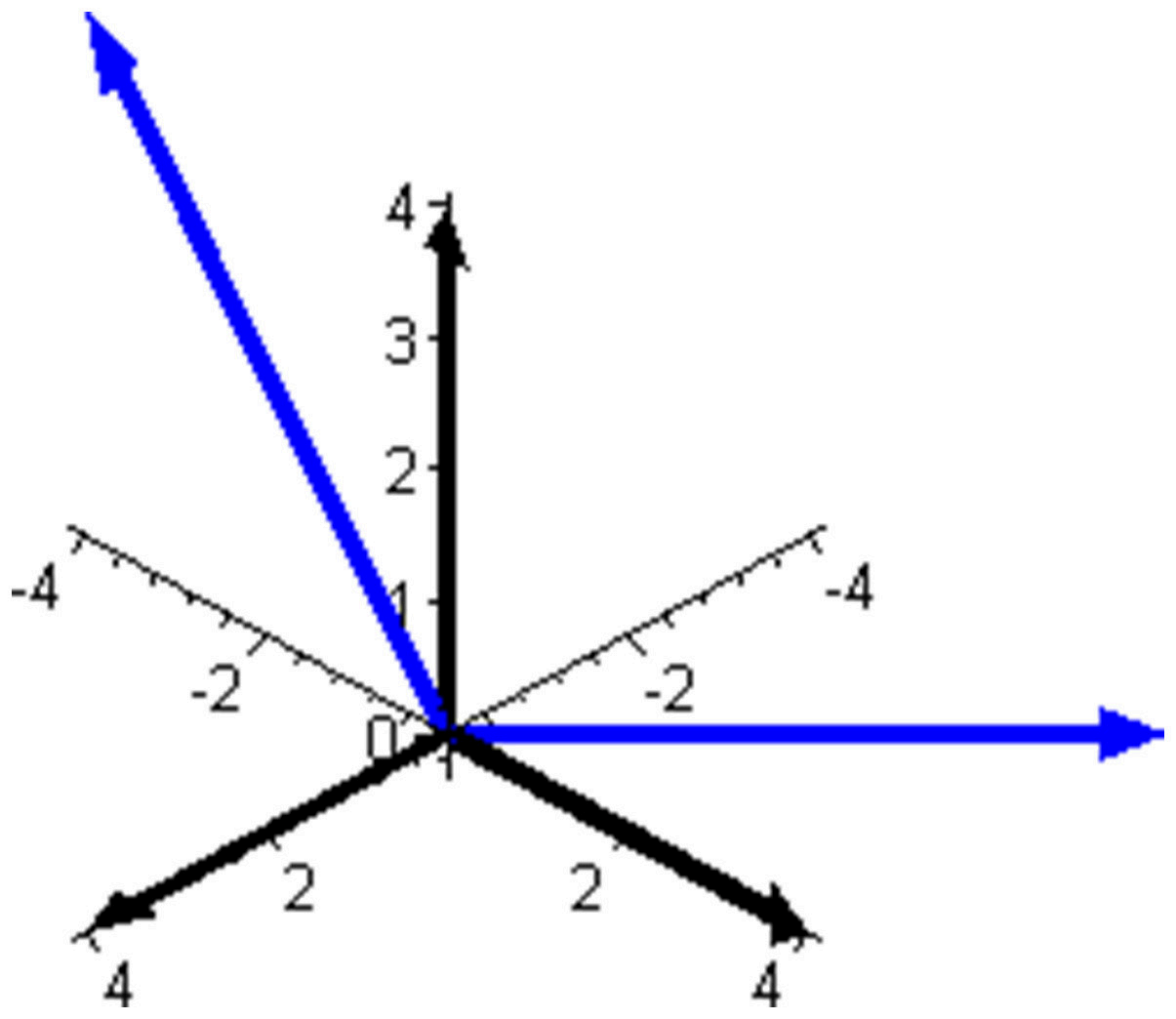
Two bases that can be used to express the reaction rate vector in the mixture of butene isomers. The basis of the component space, leading to three component rates as coordinates of **J**, is shown using black arrows. The basis of subspace V, illustrated in Figure 2 and leading to two independent reaction rates as coordinates of **J**, is shown using blue arrows.

Subspace W is one-dimensional in this mixture and is illustrated in Figure 2 together with the complementary (and orthogonal) two-dimensional subspace V. The two ways of expressing the coordinates of the reaction rate vector are illustrated in Figure 3 by examples of the two respective sets of base vectors.

The two selected independent reactions, corresponding to (B4), are 1,2-butene = cis-2,3-butene (Nr. 1) and cis-2,3-butene = trans-2,3-butene (Nr. 2). If the reaction trans-2,3-butene = 1,2-butene (Nr. 3) also occurs, then its rate is not independent. Then, the question arises of how it can be expressed in terms of the rates of the two independent reactions.

Independent and dependent reaction rates are generally different and should be strictly differentiated. In fact, as given in the introduction, reaction rates themselves cannot be measured; that is, only component rates can be measured. For the isomerization of butenes, it is sufficient to measure only two component rates, which then determine the rates of the two independent reactions, cf. (B5). If one wants to describe this isomerization in terms of all three chemically potential reactions (three traditional reaction steps), then the component rates should be transformed into a different set of rates of three reactions that are not independent. These are denoted as {*r*_**1**_, *r*_**2**_, *r*_**3**_} (the numbering in subscripts is in bold to distinguish the numbering of dependent and independent reactions). Determining the relationship between the sets of independent and dependent reaction rates is an easy task (see Supplementary Material):

(B6) $$\begin{array}{rcl} J_{1} & = & r_{1}-r_{3};\\ J_{2} & = & r_{2}-r_{3}. \end{array}$$

Equation (B6) shows that only the differences between *r*_*i*_ pairs are independent and that the set of dependent reaction rates is not made from the set of independent reaction rates simply and directly by adding an additional rate, i.e., *J*_1_ ≠ *r*_**1**_, *J*_2_ ≠ *r*_**2**_. Mathematically determined independent reaction rates thus need not be directly equal to single rates of (some) reactions occurring in the reaction scheme suggested on a chemical basis. Specific relationships between the rates *r*_**i**_ can be found if their explicit form is known; for an example of mass-action kinetics, see Pekař (2007).

It is quite common to design reaction schemes preferably on a chemical basis and not on the basis of the linear algebraic procedure described above, the latter giving only independent reactions and their rates. However, the rates of a set of dependent reactions cannot be unambiguously related to the measured experimental rates. In the example of butene isomerization, one has the following relationships.

(B7) $$\begin{array}{rcl} J^{1,2} & = & -r_{1}+r_{3}\\ J^{\text{cis}2,3} & = & r_{1}-r_{2}\\ J^{\text{trans}2,3} & = & r_{2}-r_{3} \end{array}$$

Equation (B7) is a set of algebraic equations for the transformation of measured component rates into the reaction rates of a suggested reaction scheme. The solution to this set is generally $r_{\text{1}}=-J^{1,2}+Z$, $r_{\text{2}}=-J^{1,2}-J^{\text{cis}2,3}+Z$, *r*_**3**_ = *Z*, where *Z* is an arbitrary (real) nonzero number. *Z* is also a “stationary” solution to (B7): if *r*_**1**_ = *r*_**2**_ = *r*_**3**_ = *Z*, then all component rates vanish. Such an algebraic transformation of component rates into the rates of reactions is usually not considered in kinetics. In the case of three isomerization reactions with rates expressed in the traditional mass action form, the dependence between dependent reaction rates leads to a restriction on the rate constants, known also as the detailed balance condition (Denbigh, 1951; Pekař, 2007).

Component rates are also the key to answering the question posed above, i.e. below Equation (B5). The procedure for answering this question is given in the Supplementary Material; here, we state only that it is based on the fact that component rates are still the same regardless of whether they are expressed using the triplet of dependent reactions (B7), or the pair of independent reactions (B5). The result is

(B8) $$\begin{array}{r} r_{3}=\left(a/c\right)r_{1}+\left(b/c\right)r_{2}, \end{array}$$

where *c* = 1+*a*+*b* and *a*, *b* are (arbitrary) numbers fulfilling the following matrix equation.

(B9) $$\begin{array}{r} \left[\begin{array}{ccc} -1 & 0 & 1\\ 1 & -1 & 0\\ 0 & 1 & -1 \end{array}\right]\left[\begin{array}{cc} 1+a & b\\ a & 1+b\\ a & b \end{array}\right]=\left[\begin{array}{cc} -1 & 0\\ 1 & -1\\ 0 & 1 \end{array}\right] \end{array}$$

Note that the last matrix is the transposed stoichiometric matrix (B4) and the first matrix is a similar matrix for the system of three reactions [Nr. 1–3 listed after (B5)]. Thus, expressing the dependent rate *r*_**3**_ in terms of the other two rates is not unambiguous generally. An example of expressing *a* and *b* in terms of traditional mass-action rate constants can be found in the Supplementary Material.

### Mixture of atomic and molecular oxygens

The last analyzed example (but one used in the subsequent section) is a mixture of components formed from a single atom—oxygen: O (Nr. 1), O_2_ (Nr. 2), O_3_ (Nr. 3). It can easily be found that *h* = 1 for this mixture and thus that two independent reactions are possible. The participation of an inert component can also be considered in this mixture^3^, but because it is conserved *per se* in any reaction, it has no influence on the mathematical treatment demonstrated here. If there were *I* inerts, then *h* = 1+*I* (it is self-evident that an inert is a pseudoatomic substance) and *n* = 3+*I*; consequently, *n*−*h* is always equal to 2. Pilling and Seakins (1996) give the following four reactions for this mixture (M is the inert).

(O1) $$\begin{array}{r} 1.\;{\text{O}}_{2}+\text{h}\nu =2\text{O}\\ 2.\text{ O}+{\text{O}}_{2}+\text{M}={\text{O}}_{3}+\text{M}\\ 3.\;{\text{O}}_{3}+\text{h}\nu =\text{O}+{\text{O}}_{2}\\ 4.\text{ O}+{\text{O}}_{3}=2{\text{O}}_{2} \end{array}$$

Note that from the point of view of the permanence of atoms, the second and third reactions are the forward and reversed directions of a single reaction; in other words, the studied mathematical treatment cannot take into account the effect of an inert or radiation—an inert is conserved *per se* while radiation has no atomic composition.

Let us select the following two independent reactions.

(O2) $$\begin{array}{r} 1.\;{\text{O}}_{2}=2\text{O}\\ 2.\text{ O}+{\text{O}}_{2}={\text{O}}_{3} \end{array}$$

Then, the following relationships between independent and dependent reactions are derived on the basis of component rates: *J*_1_ = *r*_**1**_−*r*_4_, *J*_2_ = *r*_**2**_−*r*_**3**_−*r*_4_. Two independent reactions mean that it is sufficient to measure only two component rates, which is a natural result of the permanence of atoms (6): 3*J*^3^+2*J*^2^+*J*^1^ = 0. Without loss of (mathematical) generality, let us replace set (O1) by set:

(O3) $$\begin{array}{r} 1.\;{\text{O}}_{2}=2\text{O}\\ 2.\text{ O}+{\text{O}}_{2}={\text{O}}_{3}\\ 3.\text{ O}+{\text{O}}_{3}=2{\text{O}}_{2} \end{array}$$

The transformation of the two measured independent component rates into the rates of dependent reactions of set (O3) is, in this reaction example, given by: $r_{1}=J^{1}/2-J^{3}/2+Z$, *r*_**2**_ = *Z*, $r_{3}=-J^{3}+Z$, where *Z* is an arbitrary (real) nonzero number, i.e., the second reaction rate can be selected arbitrarily and mass conservation will still be fulfilled, as in the case of butene isomerization. However, in the oxygen example, *Z* is not a “stationary” solution in the sense that when *r*_**1**_ = *r*_**2**_ = *r*_**3**_ = *Z*, all component rates do not vanish. The general relationship for the interdependence of dependent reaction rates is then derived, for example, as $r_{1}=J^{1}/2+\;r_{2}/2+r_{3}/2$. In terms of dependent reaction rates only, *r*_**3**_ = (*d*/*f*)*r*_**1**_+(*e*/*f*)*r*_**2**_, where *f* = 1+*d*+*e* and *d, e* are numbers fulfilling the condition given in the Supplementary Material.

### Finding the stoichiometric matrix

As stated above, condition (13) does not determine the stoichiometric matrix unambiguously. Hooyman (1961) proposed a method that should aid in the determination of the matrix. Because the stoichiometric matrix is of dimension (*n*−*h*) × *n* and has rank *n*−*h*, Hooyman suggested its representation as a matrix of two blocks (fields), one of which is the unit matrix (1):

(18) $$\begin{array}{r} ||P^{p\alpha }||=\left[\begin{array}{cc} 1 & \text{P} \end{array}\right]. \end{array}$$

Hooyman's method was designed to minimize the number of stoichiometric coefficients to be determined, i.e. to minimize the number of freely selectable coefficients. However, it is not a universal method and cannot be used in all cases; besides, condition (13) should be employed. Consider the reaction mixture of N_2_O (= 1), N_2_ (= 2), O_2_ (= 3), and O (= 4), analyzed previously (Pekař, 2009), with the composition matrix:

(19) $$\begin{array}{r} ||T_{\sigma \alpha }||\equiv ||S_{\sigma \alpha }||=\left[\begin{array}{cccc} 2 & 2 & 0 & 0\\ 1 & 0 & 2 & 1 \end{array}\right]. \end{array}$$

Hooyman's suggestion is:

(20) $$\begin{array}{r} ||P^{p\alpha }||=\left[\begin{array}{cccc} 1 & 0 & P^{13} & P^{14}\\ 0 & 1 & P^{23} & P^{24} \end{array}\right]. \end{array}$$

It can easily be checked that (13) cannot be fulfilled with this stoichiometric matrix. Even the more general form:

(21) $$\begin{array}{r} ||P^{p\alpha }||=\left[\begin{array}{cccc} 1 & P^{12} & P^{13} & P^{14}\\ 0 & 1 & P^{23} & P^{24} \end{array}\right] \end{array}$$

does not help. The problem is that although every matrix can be transformed by Gauss or Jordan elimination to upper triangular or “Hooyman's” form, respectively, not every such matrix can serve as a stoichiometric matrix. Elementary matrix transformations can be expressed in the form of matrix multiplications, but condition (13) allows for left-hand-side multiplication only, i.e. for row transformations only. Renumbering the components to 1 = O_2_, 2 = O, 3 = N_2_O, 4 = N_2_ gives:

(22) $$\begin{array}{r} ||T_{\sigma \alpha }||\equiv S_{\sigma \alpha }=\left[\begin{array}{cccc} 0 & 0 & 2 & 2\\ 2 & 1 & 1 & 0 \end{array}\right] \end{array}$$

and now (20) can be used, giving the following two independent reactions: 2N_2_O = 2N_2_ + O_2_, N_2_O = N_2_ + O. The renumbering transforms the columns of matrix (19). However, in this case, Hooyman's method does not give reactions that would correspond to experiments (Hunter, 1934), i.e. N_2_O = N_2_ + O, O + N_2_O = O_2_ + N_2_. For this reaction mixture, this can be achieved with the generalization (21), which leaves one stoichiometric coefficient for free selection. The best practical way to find a stoichiometric matrix is to combine its general form ensuring its rank with chemical (experimental) information.

### Summary discussion

The example of butene isomerization clearly illustrates two approaches to rates of reactions. One can use a mathematical procedure to determine the number of independent reactions and translate the measured component rates into reaction rates with the aid of an acceptable stoichiometric matrix. The other approach is to propose a reaction scheme using chemical considerations. However, if the scheme contains more reactions than the number of independent reactions, the measured component rates cannot be translated unequivocally into dependent reaction rates, and some reaction rates are determined by other reaction rates, anyway. In this case, one can use either a mathematically derived set of independent reactions as a “formal” reaction scheme sufficient to describe the mathematics of chemical transformation but not necessarily containing all really occurring chemical processes, or a “chemically” proposed scheme, keeping in mind that determining the rates of all its steps independently is not possible.

What are the kinetic consequences of the arbitrary selection of dependent reaction rates (one in the case of the butenes example)? Mathematics enables this selection without violating mass conservation—for example, it remains conserved for any values of *Z* defined below (B7). If there were a chemical guide (experiment) to select the proper value from all freely selectable numbers, then the arbitrariness would be removed.

What about the consequences for experiments and for the measurement and evaluation of kinetic data? Data are collected in suitable reactors; for simplicity, but without loss of generality, let us consider batch and continuously stirred tank reactors (constant volume) with balances:

(23) $$\begin{array}{r} \frac{\text{d}c_{\alpha }}{\text{d}t}=J^{\alpha } \end{array}$$

(24) $$\begin{array}{r} \frac{\text{d}c_{\alpha }}{\text{d}t}=\frac{F^{0}c_{\alpha }^{0}-Fc_{\alpha }}{V}+J^{\alpha } \end{array}$$

(the third basic system—plug flow—can be modeled as a series of continuous stirred tank reactors). Measured data are “hidden” not only in the derivatives but also in the component rates *J*^α^ together with kinetic parameters (rate constants). The equations should be solved with data to find the parameters, and for our purpose it makes no difference whether they are in nonstationary or steady-state (zero time derivatives) form.

The permanence of atoms (6) can decrease the dimensionality of this problem in the sense of decreasing the number of necessary concentrations or equations to be taken into account^2^. Let us call this “component dimensionality.” In the butene isomerization example, the permanence is expressed (rather trivially) as *J*^1, 2^+*J*^cis2, 3^+*J*^trans2, 3^ = 0; thus, only two components (and their rates) are sufficient (the component dimensionality is reduced from 3 to 2). Equation (B5) shows that the best selection should be J^1, 2^ and *J*^trans2, 3^, which also directly determines the rates of the two independent reactions (the former with reversed sign). With more complex reaction mixtures and reaction schemes (networks or mechanisms), this reduction in dimensionality need not be so trivial or clear, and an analysis based on Bowen's general results should be simpler and more straightforward than, for example, the methodology described by Al-Khateeb et al. (2009), which is, as published, limited to batch systems.

The exploitation of Equations (23) and (24) requires explicit expressions of *J*^α^ as functions of concentrations. Of course, these are not delivered by Bowen's approach analyzed here, not even after their “translation” into the reaction rates *J*_*p*_. Here, traditional kinetic mass-action law and expressions are usually used. If we select, for some chemical reasons, more reaction steps (and their rates*r*_*j*_) than the number of independent reactions, then the (in)dependence places some restrictions on the values of their parameters, i.e., rate constants; specific examples can be found in Pekař (2007, 2016). Then, dimensionality in the sense of the number of (selectable) mass-action kinetic parameters—which can be called “parametric dimensionality”—is effectively reduced. The existence of a limited number of independent reactions itself need not reduce parametric dimensionality, as was illustrated by butene isomerization. In the context of this example, the existence of only two independent reactions does not mean that we can simply work with only two members of the triplet {*r*_**1**_, *r*_**2**_, *r*_**3**_}; indeed, the independent reactions are the differences *r*_**1**_−*r*_**3**_, *r*_**2**_−*r*_**3**_. Another possibility is to express the independent reactions *J*_*p*_ as functions of concentrations in terms of the thermodynamic polynomial (for details and examples see Pekař, 2009; Pekař and Samohýl, 2014), which develops Bowen's approach to practical kinetic applications and directly reduces parametric dimensionality. This is because this polynomial does not work with rate constants in the reversed direction and uses equilibrium constants of independent reactions, which can be, in principle, calculated from thermodynamic databases independently of kinetics.

Linear algebraic treatment enables us to find relationships between dependent and independent reactions; as an example, see (B8). However, they are rather general and not unambiguous, as follows from the elementary results of linear algebra. On the other hand, unambiguous forms can be found for specific (mass-action) cases, as mentioned above. A similar attempt by Vlad and Ross (2009) to express dependent rates using independent rates resulted only in the expression of the ratio of forward and backward rates of dependent reactions in terms of ratios of forward and backward rates of independent reactions, and is ambiguous because the same ratio (fraction) can be obtained for an unlimited number of forward and backward ratios (combinations of the numerator and denominator). Their work is valid for mass-action kinetic expressions only. Thus, the linear algebraic approach presented here really seems to be a step toward a general treatment of the issue of (in)dependent reactions.

Both of the works cited above (Al-Khateeb et al., 2009; Vlad and Ross, 2009) are examples of an “*a posteriori*” analysis of dependencies among reactions. This means that a reaction scheme is proposed first and only then is the (in)dependence evaluated. Bowen's approach is of an “*a priori*” type; no reactions are necessary but only the components of a reaction mixture. The number and set of independent reactions then follow naturally while the space for modifications based on chemical insights is still open. This approach seems to be more natural and consistent, especially when (chemical) data are to be evaluated mathematically.

For the sake of simplicity and illustration, simple reacting mixtures were used as examples. Subsequent work on mixtures containing tens or hundreds of reacting species is going on to analyze the impacts of the presented methodology on more complex (and realistic) systems. Further, the principle of charge neutrality may provide additional constraints in ionic reacting systems; this issue is beyond the scope of Bowen's method and this work.

## Conclusions

The existence of reaction rates is a mathematical consequence of mass conservation (in other words, the permanence of atoms). Linear algebra shows how (measurable) component rates can be translated elegantly and effectively into rates of individual reactions. Mathematical analysis starts just with the list of components of a reaction mixture; it thus requires very basic and elementary input information and operates with independent reactions only. The latter can be identified with the steps of a reaction scheme proposed on the basis of chemical experimentation and insight; chemistry can thus help to resolve certain ambiguities inherent in the general mathematical treatment.

Often chemistry suggests more reaction steps than the number of independent reactions. Reaction rates in such schemes are not independent; some of them can be expressed using the remaining ones, and a set of such reaction rates cannot be unambiguously related to measured component rates. Even if the same reaction occurs both in the (“mathematical”) set of independent reactions and in the (“chemical”) scheme containing dependent reactions, its rate can be different in these two cases.

This apparent paradox is a consequence of the effort to describe the mathematics of chemical transformations by more reactions than is necessary. Chemistry may simply add more reactions (reaction steps) than is necessary mathematically and is dictated by mass conservation. The paradox can be resolved by using only independent reactions and their rates in the mathematical data treatment or reactor design; that is, when just mathematics is used, and discussing the whole scheme when just chemistry (a description of reaction events on the microscopic, i.e., atomic and molecular, level) is the primary aim. Mathematically, it is not necessary to deal with or analyze rates of dependent reactions because they are determined by the rates of the other reactions.

In this way, the dimensionality of the data evaluation problem can be reduced, both from the point of view of the number of components concentrations that should be measured and from the point of view of the number of necessary kinetic parameters.

## Author contributions

The author confirms being the sole contributor of this work and approved it for publication.

### Conflict of interest statement

The author declares that the research was conducted in the absence of any commercial or financial relationships that could be construed as a potential conflict of interest.

## Figures and Tables

**Table:** 

*A*^σ^	Atomic weight of atom σ
α	Component of reaction mixture
*c*_α_	Molar concentration of component α
**d**^*p*^	Basis of subspace V
${\text{e}}_{\alpha },\;{\text{e}}^{\alpha }$	Basis vectors
*E*^σ^	Weight of pseudoatomic element σ
**f**_σ_	Basis of subspace W
*F*	Volumetric flow rate
**J**	Reaction rate vector
*J*^α^	Molar production rate (per unit volume and unit time) of component α
*J*_*p*_	Rate of independent reaction *p*
**M**	Vector of molar masses
*M*_α_	Molecular weight of component α
*P*^*pα*^	Stoichiometric coefficient of component α in reaction *p*; element of stoichiometric matrix
*r*_***i***_	Rate of (dependent) reaction ***i***
*r*_*wα*_	Mass production rate (per unit volume and unit time) of component α
*T*_σα_	Number of atoms σ in component α
*t*	Time
*V*	Reactor volume
^0^	Superscript indicating reactor inlet

## Footnotes

^1^An example, butene isomerization, is given below.

^2^This was realized, e.g., by Missen et al. (1999) who state that stoichiometry gives the minimum number of species to be analyzed.
