# Supplementary material for: Rates of Reactions as a Mathematical Consequence of the Permanence of Atoms and the Role of Independent Reactions in the Description of Reaction Kinetics
Source: Front Chem. 2018 Jul 27;6:287. doi: 10.3389/fchem.2018.00287 (PMC6073800; doi:10.3389/fchem.2018.00287)
Supplement: Supplementary file 1 [file Data_Sheet_1.PDF]

frontiers

# *Supplementary Material*

# Rates of reactions as a mathematical consequence of the permanence of atoms and the role of independent reactions in the description of reaction kinetics

**Miloslav Pekař ***

*** Correspondence:** Corresponding Author: pekar@fch.vut.cz

**Derivation of Eq. (B6).** Component rates can be expressed in two alternative ways – either with the independent or dependent reaction rates[1]:

$$[J^{1,2} \quad J^{\mathrm{cis}2,3} \quad J^{\mathrm{trans}2,3}] = [J_1 \quad J_2]\begin{bmatrix} -1 & 1 & 0 \\ 0 & -1 & 1 \end{bmatrix} = [r_1 \quad r_2 \quad r_3]\begin{bmatrix} -1 & 1 & 0 \\ 0 & -1 & 1 \\ 1 & 0 & -1 \end{bmatrix}.$$

Performing matrix multiplication, we obtain:

$$[-J_1 \quad J_1 - J_2 \quad J_2] = [-r_1 + r_3 \quad r_1 - r_2 \quad r_2 - r_3],$$

from which (B6) follows.

**Derivation of Eq. (B8).** Similarly as above, let us express the component rates in two alternative ways:

(i) using independent reaction rates:

$$\begin{bmatrix} J^{1,2} \\ J^{\mathrm{cis}2,3} \\ J^{\mathrm{trans}2,3} \end{bmatrix} = \begin{bmatrix} -1 & 0 \\ 1 & -1 \\ 0 & 1 \end{bmatrix}\begin{bmatrix} J_1 \\ J_2 \end{bmatrix} \equiv \|P\| \begin{bmatrix} J_1 \\ J_2 \end{bmatrix} \qquad \text{(S1)}$$

(ii) using dependent reaction rates:

[1] The numbering of reaction is 1,2-butene = cis-2,3-butene (Nr. 1), cis-2,3- butene = trans-2,3-butene (Nr. 2), trans-2,3-butene =1,2-butene (Nr. 3); cf. the main text below Eq. (B5).

$$\begin{bmatrix} J^{1,2} \\ J^{\mathrm{cis}2,3} \\ J^{\mathrm{trans}2,3} \end{bmatrix} = \begin{bmatrix} -1 & 0 & 1 \\ 1 & -1 & 0 \\ 0 & 1 & -1 \end{bmatrix} \begin{bmatrix} r_1 \\ r_2 \\ r_3 \end{bmatrix} \equiv \|P'\| \begin{bmatrix} r_1 \\ r_2 \\ r_3 \end{bmatrix}. \qquad \text{(S2)}$$

The transformation from matrix $\|P\|$ to matrix $\|P'\|$ is unambiguous

$$\|P'\| = \|P\| \begin{bmatrix} 1 & 0 & -1 \\ 0 & 1 & -1 \end{bmatrix} \qquad \text{(S3)}$$

in contrast to the reversed transformation (simply because $\|P'\|$ is the stoichiometric matrix of the set of dependent reactions):

$$\|P\| = \|P'\| \begin{bmatrix} 1+a & b \\ a & 1+b \\ a & b \end{bmatrix}$$

where the numbers $a$ and $b$ can evidently be selected arbitrarily.

Eq. (S1) can be transformed

$$\|P\| \begin{bmatrix} J_1 \\ J_2 \end{bmatrix} = \|P'\| \begin{bmatrix} 1+a & b \\ a & 1+b \\ a & b \end{bmatrix} \begin{bmatrix} J_1 \\ J_2 \end{bmatrix} = \|P'\| \begin{bmatrix} (1+a)J_1 + bJ_2 \\ aJ_1 + (1+b)J_2 \\ aJ_1 + bJ_2 \end{bmatrix}. \qquad \text{(S4)}$$

Comparing (S1) and (S2) with (S4) and substituting from (B6),

$$\begin{aligned} J_1 &= r_1 - r_3 \\ J_2 &= r_2 - r_3, \end{aligned} \qquad \text{(B6)}$$

we obtain

$$\begin{gathered} r_3 = aJ_1 + bJ_2 = a(r_1 - r_3) + b(r_2 - r_3) = ar_1 + br_2 + (-a-b)r_3 \Rightarrow \\ r_3 = [a/(1+a+b)]r_1 + [b/(1+a+b)]r_2 \end{gathered}$$

which, with $c = 1 + a + b$, gives (B8) immediately.

Note, that the matrix

$$\begin{bmatrix} 1 & 0 & -1 \\ 0 & 1 & -1 \end{bmatrix}$$

in (S3) shows that the dependent reaction C = A is the sum of negative (reversed) independent reactions, –(A = B) – (B = C)[§]. However, it does not follow that $r_3 = -r_1 - r_2$.

**Mass-action example to Eq. (B8).** It was shown in Pekař (2007) that when dependent rates are expressed by the traditional mass-action law:

$$r_1 = k_1 c_A - k_2 c_B;\ r_2 = k_3 c_B - k_4 c_C;\ r_3 = k_5 c_C - k_6 c_A.$$

then $r_3 = -(k_6/k_1) r_1 - (k_5/k_4) r_2$. Consequently, $a/c = -(k_6/k_1)$ and $b/c = -(k_5/k_4)$. From these expressions, the parameters $a$ and $b$ can be expressed in (rather complex) terms of the rate constants ($k_i$).

**Atomic and molecular oxygens mixture, set (O3).** The component rates can be expressed as

$$\begin{bmatrix} J^1 \\ J^2 \\ J^3 \end{bmatrix} = \begin{bmatrix} 2 & -1 \\ -1 & -1 \\ 0 & 1 \end{bmatrix} \begin{bmatrix} J_1 \\ J_2 \end{bmatrix}$$

or as

$$\begin{bmatrix} J^1 \\ J^2 \\ J^3 \end{bmatrix} = \begin{bmatrix} 2 & -1 & -1 \\ -1 & -1 & 2 \\ 0 & 1 & -1 \end{bmatrix} \begin{bmatrix} r_1 \\ r_2 \\ r_3 \end{bmatrix}.$$

By a similar procedure as above with (S1)-(S4) we arrive at the condition for the (arbitrary) numbers $d$ and $e$ in $r_3 = (d/f) r_1 + (e/f) r_2$:

$$\begin{bmatrix} 2 & -1 & -1 \\ -1 & -1 & 2 \\ 0 & 1 & -1 \end{bmatrix} \begin{bmatrix} 1+d & e \\ d & 1+e \\ d & e \end{bmatrix} = \begin{bmatrix} 2 & -1 \\ -1 & -1 \\ 0 & 1 \end{bmatrix}.$$

## References


Pekař, M. (2007). Detailed balance in reaction kinetics – consequence of mass conservation? React. Kinet. Catal. Lett. 90, 323-329.


[§] For the sake of simplicity and generality, butene isomers are denoted here as A, B, and C.
